# Effect of N-Vinyl-2-Pyrrolidone (NVP), a Bromodomain-Binding Small Chemical, on Osteoblast and Osteoclast Differentiation and Its Potential Application for Bone Regeneration

**DOI:** 10.3390/ijms222011052

**Published:** 2021-10-13

**Authors:** Viviane A. Klemmer, Nupur Khera, Barbara M. Siegenthaler, Indranil Bhattacharya, Franz E. Weber, Chafik Ghayor

**Affiliations:** 1 Oral Biotechnology and Bioengineering, Center for Dental Medicine, University of Zurich, 8032 Zurich, Switzerland; viviane.klemmer@uzh.ch (V.A.K.); nupur.khera@ki.se (N.K.); barbara.m.siegenthaler@gmail.com (B.M.S.); Indranil.Bhattacharya@usz.ch (I.B.); 2 Center for Applied Biotechnology and Molecular Medicine, University of Zurich, 8057 Zurich, Switzerland

**Keywords:** BMP2, osteoblast, osteoclast, bone regeneration, bromodomain inhibitor, small chemical

## Abstract

The human skeleton is a dynamic and remarkably organized organ system that provides mechanical support and performs a variety of additional functions. Bone tissue undergoes constant remodeling; an essential process to adapt architecture/resistance to growth and mechanical needs, but also to repair fractures and micro-damages. Despite bone’s ability to heal spontaneously, certain situations require an additional stimulation of bone regeneration, such as non-union fractures or after tumor resection. Among the growth factors used to increase bone regeneration, bone morphogenetic protein-2 (BMP2) is certainly the best described and studied. If clinically used in high quantities, BMP2 is associated with various adverse events, including fibrosis, overshooting bone formation, induction of inflammation and swelling. In previous studies, we have shown that it was possible to reduce BMP2 doses significantly, by increasing the response and sensitivity to it with small molecules called “BMP2 enhancers”. In the present study, we investigated the effect of N-Vinyl-2-pyrrolidone (NVP) on osteoblast and osteoclast differentiation in vitro and guided bone regeneration in vivo. We showed that NVP increases BMP2-induced osteoblast differentiation and decreases RANKL-induced osteoclast differentiation in a dose-dependent manner. Moreover, in a rabbit calvarial defect model, the histomorphometric analysis revealed that bony bridging and bony regenerated area achieved with NVP-loaded poly (lactic-co-glycolic acid (PLGA) membranes were significantly higher compared to unloaded membranes. Taken together, our results suggest that NVP sensitizes BMP2-dependent pathways, enhances BMP2 effect, and inhibits osteoclast differentiation. Thus, NVP could prove useful as “osteopromotive substance” in situations where a high rate of bone regeneration is required, and in the management of bone diseases associated with excessive bone resorption, like osteoporosis.

## 1. Introduction

N-Vinyl-2-pyrrolidone (NVP) is the main monomer used in the production of polyvinylpyrrolidone (PVP), a versatile polymer better known as povidone [1]. PVP is a water-soluble polymer used in applications and fields as diverse as the petroleum industries, cosmetics and adhesives. Additionally, PVP is a component/drug approved by the U.S Food and Drug Administration (FDA). Historically, in the 1940s, PVP was used as a plasma volume expander [2]. However, nowadays PVP is used as a binder in many pharmaceutical tablets, as a lubricant in some contact lenses and their packing solution as well as in some eye drops [3]. PVP is also used in personal care products, such as shampoos and toothpastes. As a food additive, PVP is a stabilizer and has the E number E1201 [4]; it is used in the wine industry as a fining agent for white wine and some beers. The FDA has approved PVP for many uses and it is generally considered safe [3]. In this context, the biological effects of PVP are well documented throughout the literature [5], however, the effects of its monomer NVP are not deeply studied and fully understood in particular in bone biology.

Bone is a dynamic and well-organized tissue that provides mechanical support and performs a variety of functions, such as calcium homeostasis [6]. Bone tissue undergoes constant remodeling; a process that involves the resorption of mineralized bone followed by the formation of a new bone matrix to preserve skeletal integrity. The interaction, differentiation and functions of two main cell lines present in the bone tissue are essential for this process: the mesenchymal osteoblastic lineage and the hematopoietic osteoclastic lineage [7].

In previous studies, it has been shown that N-methyl-2-pyrrolidone (NMP), which has a chemical structure similar to NVP, increases bone regeneration and prevents estrogen depletion-induced osteoporosis [8]. Furthermore, NMP decreases osteoclastic differentiation and bone resorption activity making this compound a good candidate for new therapeutic approaches for the management of osteoporosis and compromised bone healing conditions [9,10].

From a mechanistic point of view, NMP acts as a bromodomain inhibitor and thus participates in the epigenetic regulation processes [8]. Epigenetics describes the mechanisms that control gene expression without changing the DNA sequence. One of the most studied epigenetic mechanisms is the histone modifications and principally the acetylation of lysine residues in the N-terminal tails of histone proteins [7].

The bromodomain and extra-terminal domain (BET) family of proteins (composed of BRD2, BRD3, BRD4 and BRDT “testis specific”) is a small family of proteins that recognize and bind to acetylated lysine residues on tails histone, and therefore are considered “readers” of the histone code. Bromodomain is a structural motif of 110 amino acids that form a bundle of four helices (αZ, αA, αB and C) where the interhelical loops αZ-αA (ZA) and αB-αC (BC) create a hydrophobic pocket that recognizes acetyl-lysine modifications [11]. Their association with chromatin plays an important role in cell development [12], and inhibition of bromodomains has already been discussed as promising therapeutic for multiple diseases [13]. The discovery of the drugability of bromodomains has fueled the development of small molecule inhibitors [14,15]. In our group, we showed that NMP acts as a bromodomain inhibitor and plays a role as an epigenetic regulator [8].

Bromodomain inhibitors like NMP mimic acetylated lysine and bind to bromodomains, thus disturbing the binding of these domains to histones and affecting chromatin structure and gene transcription.

Since the chemical structures of NMP and NVP are quite similar, it suggests that NVP has a comparable impact on bone cells as NMP. To explore this hypothesis, we evaluated the toxicity of NVP, its effect on osteoblast and osteoclast differentiation in vitro as well as on bone regeneration in vivo in an animal model of bone defect.

## 2. Results

Guided bone regeneration (GBR) is a method commonly used in dentistry to restore alveolar deficiencies for the placement of dental implants [16]. Initially, the non-resorbable polytetrafluorethylene (ePTFE) membranes, and lately the resorbable native collagen membranes, are used most frequently [17]. In the course of the development of a biodegradable GBR membrane, we performed in vivo studies to screen various prototypes [18,19]. We reported that GBR membranes treated in particular with NMP induced an acceleration of bone regeneration in a rabbit calvarial defect model. NMP has a long track record as a constituent in FDA-approved medical devices and presents a chemical structure similar to NVP (Figure S1). Considering the results obtained with NMP, we decided to evaluate the effect of NVP on bone regeneration.

### 2.1. NVP Enhances BMP2-Induced Osteoblast Differentiation in C2C12 Cells

**Figure 1:**
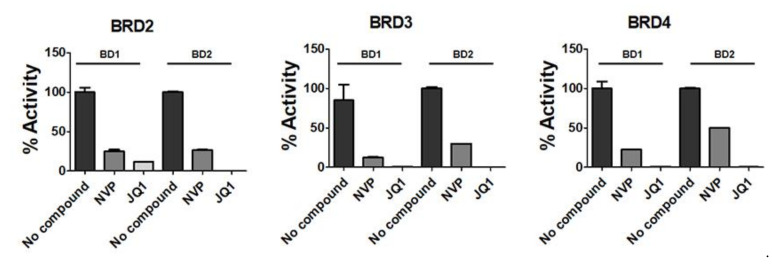
NVP inhibits the binding activity of bromodomains: effects of 10 mM NVP on the binding ability of recombinant BD1 and BD2 domains of BRD2, BRD3 and BRD4 using the AlphaScreening assay (*n* = 4). JQ1 (1 μM) was used as a prototype of bromodomain inhibitor.

In our previous studies, we showed that a large portion of the bioactivity of NMP could be related to its affinity to bromodomains and its role as a bromodomain inhibitor [8,20,21]. To test NVP for its affinity to bromodomains, we employed an AlphaScreen assay and studied the effect of NVP on the binding of different human bromodomains (Figure 1). We observed that NVP significantly inhibits the binding activity of BRD2, BRD3 and BRD4. Compared to the high-affinity bromodomain inhibitor JQ1 (μM range), the NVP concentrations needed for bromodomain inhibition are in the mM range. Despite this difference, the effect of NVP should be visible both in vitro and in vivo as has been demonstrated for NMP also considered as “low affinity” bromodomain inhibitor.

**Figure 2:**
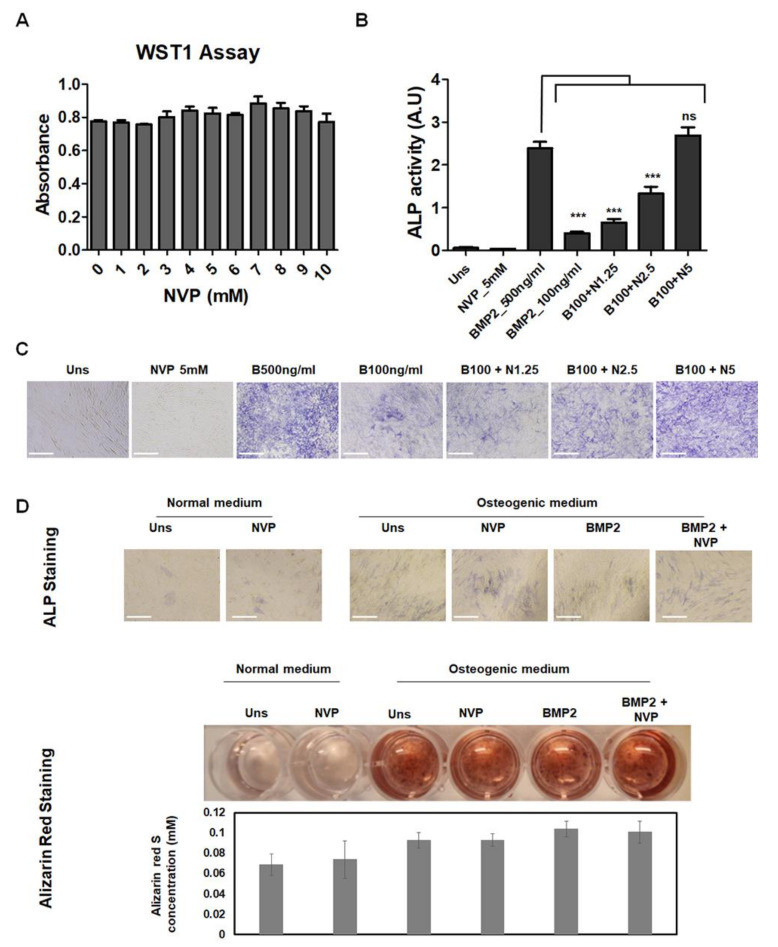
The effect of NVP on the viability and BMP2-induced osteoblast differentiation. (**A**) C2C12 cytotoxicity assay using the WST-1 reagent. The absorbance (450–600 nm) directly correlates to cell number. (**B**,**C**) Effects of NVP on BMP-induced Alkaline Phosphatase Activity (ALP) in C2C12 cell line. Cells were treated with BMP2 (500 or 100 ng/mL), or BMP2 and NVP (B+N) at the concentration indicated ranging from 1.25 to 5 mM. After 6 days, the cells were lysed to measure ALP activities (**B**) or fixed and stained for ALP (**C**) as described in Materials and Methods. Error bar indicates S.D. from triplicate samples. For BMP2 (500 ng/mL) vs. BMP2 (100 ng/mL) with or without NVP ***: *p* < 0.001. (**D**) Effects of NVP on ALP and calcium deposition in primary osteoblasts. Rabbit primary osteoblast were seeded in 24-well culture plates in normal medium (alpha-MEM, 10%FBS) or osteogenic medium ((alpha-MEM, 10%FBS, 50 µg/mL ascorbic acid, 10 mM β-glycerophosphate, 10 nM dexamethasone) and treated with NVP (5 mM), BMP2 (100 ng/mL) or BMP2 in the presence of NVP (BMP+NVP). The medium was changed every 3 days and after 6 days (for ALP) or 14 days of cultures (for Alizarin Red), cells were fixed and staining for ALP and calcium deposition respectively. Scale bar: 50 μm.

To investigate the role of NVP on bone cells, we first evaluated its effect on the viability of C2C12 cells. C2C12 mouse cells are a standard model cell-line used for the analysis of osteoblast differentiation from mesenchymal stem cells. The viability/toxicity was examined by monitoring the absorbance of formazan produced after the addition of WST-1 reagent. As shown in the Figure 2A, after 48 h of treatment with concentrations ranging from 1 to 10 mM, we did not observe any deleterious effect on cell viability. The osteogenic effects of NVP on C2C12 cells were then examined by ALP activity and ALP staining (Figure 2B,C). As already established, BMP2 redirects the differentiation of C2C12 into that of the osteoblastic lineage [22]. As shown in Figure 2B, NVP enhanced BMP2-induced ALP activity in a dose-dependent manner. The co-stimulation with 5 mM of NVP together with 100 ng/mL of BMP2 is as effective as 500 ng/mL of BMP2, suggesting that NVP amplifies the responsiveness of C2C12 cells to BMP2 by at least 5-fold increase. The ALP staining experiment showed in Figure 2C confirmed qualitatively this observation. We also performed experiment with primary osteoblasts isolated from rabbit calvaria. There is a trend that NVP enhances the effect of BMP2 particularly as seen in the ALP staining, but the result is not as significant as that obtained with the C2C12 cell line (Figure 2D). It is very likely that the effect of NVP depends on the stage of cell differentiation. Hence, in pluripotent cells such as C2C12 cells (that do not produce endogenously BMP2), NVP increases the response of cells to BMP2 and therefore promote their commitment to the osteoblastic lineage. In the study with primary osteoblasts, which are well-differentiated cells and endogenously produce BMP2, the effect of NVP on these cells is minimal.

**Figure 3:**
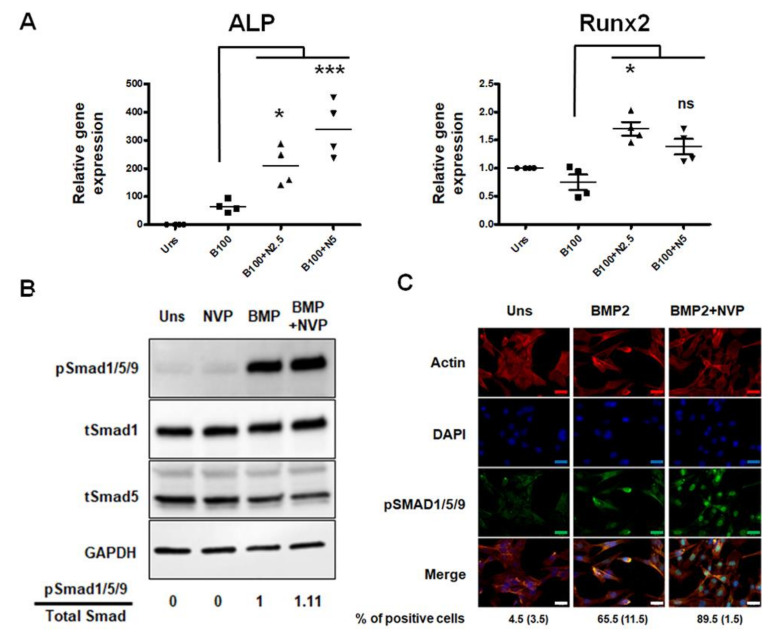
Effect of NVP on BMP2-induced osteoblast differentiation and Smad signaling. (**A**) C2C12 cells were stimulated for 48 h with BMP2 (100 ng/mL), or BMP2 and 2.5 mM or 5 mM NVP (B100+N2.5 or B100+N5). Total RNA was extracted and relative gene expression of ALP and Runx2 were analyzed by real-time PCR, normalized to Rps18 and presented as the relative level to unstimulated cells (Uns). (**B**) C2C12 cells were treated with NVP (5 mM), BMP2 (100 ng/mL) or BMP2+NVP for 30 min. Cells were lysed and total proteins were subjected to SDS-PAGE and blotted onto PVDF membrane. Phospho-Smad1/5/9 and total Smad1 were immunodetected usding specific rabbit polyclonal antibodies. GAPDH was used as a sample loading control. (**C**) C2C12 cells were grown on 15 μ-Chamber 12 well (Ibidi, Martinsried, Germany), stimulated with BMP2 (100 ng/mL) or BMP2 after 5 mM NVP pre-treatment (BMP2+NVP). After 30 min stimulation, cells were fixed and the nuclear translocation of phosphorylated Smad1/5/9 was shown by immunofluorescence staining. Error bar indicates S.D. from triplicate samples. *: *p* < 0.05; ***: *p* < 0.001. Scale bar: 25 μm.

In our cell line model, pretreatment with NVP before stimulation with BMP2 improves not only the expression of ALP mRNA but also that of Runx2, a master transcription factor for osteoblast differentiation (Figure 3A), suggesting that NVP treatment may improve the BMP2-induced osteoblastic differentiation.

Next, in order to elucidate the mechanism by which NVP influences the effect of BMP2, Smad1/5/9, a key signaling molecule driving osteogenesis, was examined. Analysis of Smad1/5/9 phosphorylation shows that there is no striking difference that could be attributed to the presence of NVP besides a slight increase in BMP2 induced Smad1/5/9 phosphorylation in presence of NVP (Figure 3B). However, the effect of NVP is obvious when the nuclear translocation of phosphorylated Smad1/5/9 was visualized (Figure 3C). In C2C12 cells exposed to BMP2, phosphorylated Smad1/5/9 was found in the cell’s nuclei. However, no nuclear phosphorylated Smad1/5/9 was seen in the unstimulated cells. The concentration of phosphorylated Smad1/5/9 in the nuclei induced by BMP2 was enhanced by NVP. These results suggest that NVP modulates BMP2/Smad signaling, slightly at the level of Smad phosphorylation but further more pronounced when it comes to the translocation into the nucleus.

### 2.2. NVP Inhibits RANKL-Induced Osteoclast Differentiation

The human skeleton undergoes continuous bone remodeling throughout life. This process involves two distinct stages, bone resorption and bone formation. As seen above, NVP can promote the differentiation of osteoblasts, the cells responsible for bone formation. In this context, we wanted to know what could be the effect of NVP on the differentiation of osteoclasts, cells which are responsible for resorption and therefore for bone degradation. To investigate that, we used bone marrow-derived monocytes (BMMs) as well as RAW264.7 cells, which provide a valuable model for studying and dissecting the osteoclast differentiation process.

**Figure 4:**
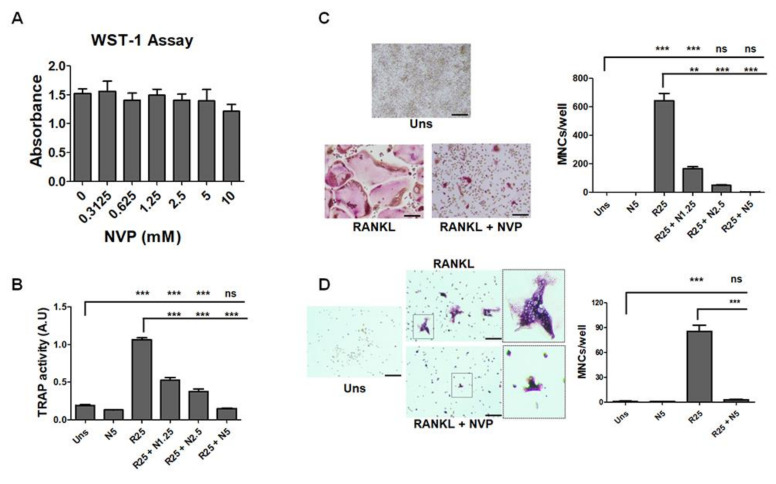
Effect of NVP on the viability and RANKL-induced osteoclast differentiation. (**A**) Cell viability. RAW264.7 cells were seeded on a 96-well plate, treated for 48 h as indicated in the figure. Cell viability/cytotoxicity was measured using WST-1 reagent as described in Materials and Methods. Data are expressed as mean ± S.D. (*n* = 4) from a representative experiment. (**B**,**C**) Effects of NVP on RANKL-induced osteoclast differentiation. RAW264.7 cells were seeded in 12-well culture plates and treated with RANKL (25 ng/mL), or RANKL in the presence of an increasing concentration of NVP ranging from 1.25 to 5 mM. After 6 days, the cells were used to measure TRAP activity (**B**) or fixed and stained for TRAP to visualize TRAP-positive multinucleated cells (MNCs) (**C**) as described in Materials and Methods. (**D**) Effect of NVP on primary cells. Bone marrow-derived monocytes (BMMs) isolated from rabbit femurs were treated with RANKL (25 ng/mL) or RANKL and NVP (5 mM) in the presence of M-CSF (25 ng/mL) for 6 days. After culturing, the cells were fixed and stained for TRAP. Stained cells were photographed, and TRAP-positive MNCs containing three or more nuclei were counted as osteoclasts. Data are expressed as the mean ± S.D. (*n* = 4). **: *p* < 0.01; ***: *p* < 0.001. Scale bar: 50 μm.

**Figure 5:**
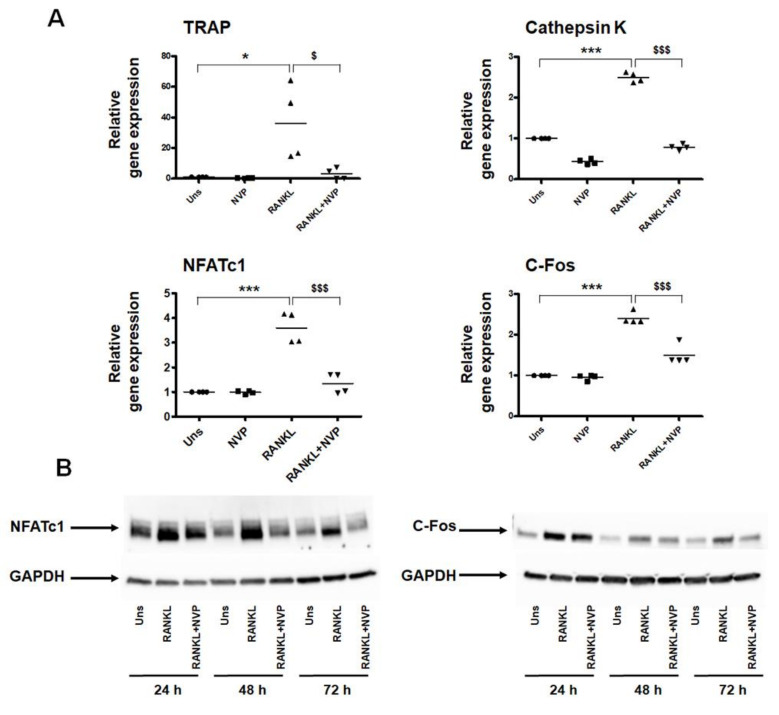
Effect of NVP on osteoclast markers. (**A**) RAW264.7 cells were stimulated for 72 h with RANKL (25 ng/mL), or RANKL in the presence of 5 mM of NVP (RANKL+NVP) as indicated. Total RNA was extracted and expression of TRAP, cathepsin K, NFATc1 and c-Fos were analyzed by real-time PCR, normalized to Rps18 and presented as the relative level to unstimulated cells (Uns). Data are expressed as the mean ± S.D (*n* = 4). Symbols (*, $) indicates statistical significance difference at *p* < 0.05, symbols (***, $$$) indicates statistical significance difference at *p* < 0.001. (**B**) Representative Western blot analysis showing NFATc1 and c-Fos expression after 24 h, 48 h and 72 h stimulation with RANKL (25 ng/mL), or RANKL in the presence of 5 mM of NVP (RANKL+NVP).

We evaluated the effect of NVP on the viability/toxicity of RAW264.7 cells, and as presented in Figure 4A, none of the concentrations used showed a detrimental effect of NVP. Interestingly, NVP inhibits RANKL-induced osteoclast differentiation in a concentration-dependent manner as measured by tartrate-resistant acid phosphatase (TRAP) activity (Figure 4B) and TRAP mRNA expression (Figure 5A). In addition, NVP appears to inhibit RANKL-induced osteoclast maturation since, in the presence of this small chemical, a lower number of giant multinucleated cells (MNCs) formed, as quantified microscopically (Figure 4C). Moreover, in the presence of NVP (1.25 mM), the number of MNCs induced by RANKL was reduced by ~75%, whereas treatment with NVP at 5 mM completely abolished the formation of MNCs. In the following experiment, we used primary bone marrow-derived monocytes (BMMs). BMMs were isolated from rabbit femurs and differentiated towards osteoclast lineage by M-CSF and RANKL treatment. As shown in Figure 4D, RANKL (with M-CSF) induced osteoclast differentiation and NVP treatment, as we saw with the RAW 264.7 cell line, inhibits RANKL-induced osteoclasts differentiation. NVP probably inhibits the monocyte fusion step as shown in the enlarged image where we see that the osteoclasts formed in the presence of NVP have a very low number of nuclei.

The bone resorption-related enzyme cathepsin K is highly expressed in osteoclastic cells and plays an important role in bone remodeling by degrading Type I collagen [23]. Therefore, we investigated the effect of NVP on the cathepsin K mRNA expression. Figure 5A demonstrates that RANKL induced an increase in cathepsin K mRNA expression and this effect was completely suppressed by the presence of NVP. It is well known that the transcription factors NFATc1 and c-Fos are essential for osteoclast differentiation [24,25,26], and as we showed earlier both are important targets for the inhibitory effect of NMP on osteoclast differentiation [10]. Stimulation of RAW264.7 cells with RANKL induced an increased expression of NFATc1 and c-Fos mRNA (Figure 5A). Treatment with NVP at 5 mM significantly decreases the mRNA expression of NFATc1 and c-Fos induced by RANKL. As shown in Figure 5B, protein expression correlated with mRNA expression. Indeed, NVP treatment significantly reduced the levels of NFATc1 and c-Fos protein expressions. After 72 h treatment, the level of the transcription factors was similar to that in unstimulated cells.

### 2.3. The Release of NVP from Loaded Membranes

**Figure 6:**
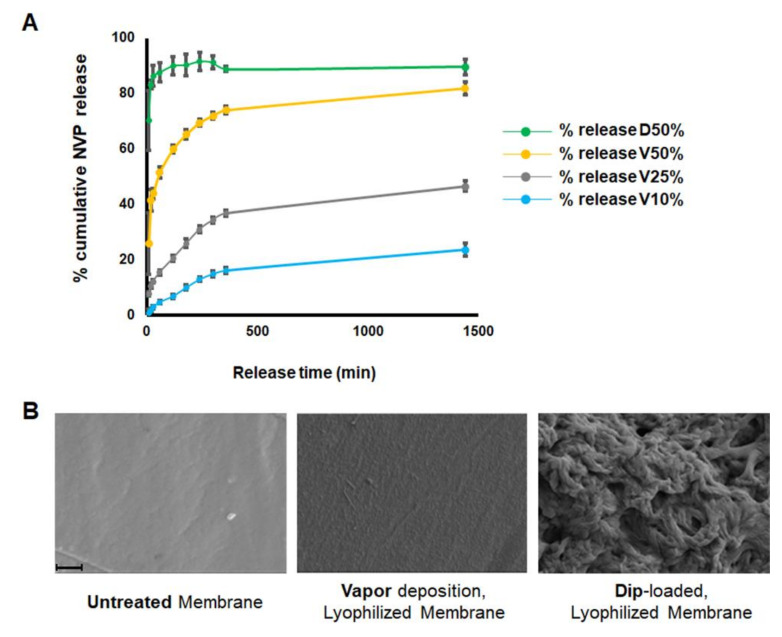
NVP release and the structure/integrity of loaded membranes. (**A**) In vitro release of NVP from membranes. Total release of the entirely loaded chemical into the PBS solution is referred to as 100% release. Individual values for each time point are represented as a percentage of “cumulative release”. “**D**” stands for dip loading and “**V**” stands for vapor deposition. (**B**) SEM images of Untreated and NVP-loaded membranes either by 10% vapor deposition or by dipping. Magnification 20,000×. Scale bar: 1 μm.

Membranes are an essential component in guided bone regeneration procedures. Several studies have shown that the bioactivity of membranes could be improved by incorporating either DNA or RNA fragments [27]. Interesting results have been also shown by modifying their physicochemical characteristics with chemicals [18,28]. Recently, we showed that NMP and N,N-dimethylacetamide (DMA), FDA approved solvents and used as drug-delivery vehicles, can be loaded onto poly-lactide-co-glycolide (PLGA) membranes, thereby increasing their ability to induce bone regeneration [19,28,29]. This was mainly due to the broad spectrum of bioactivities of NMP and DMA, which includes, among other things, enhancement of bone morphogenetic protein (BMP) activity. To investigate the effect of NVP on bone formation, we employed the same strategy. Therefore, we first studied the uptake and the release of NVP from the membranes either after vapor deposition or by dipping the membrane in pure NVP. While the dip-loaded membrane released almost all NVP within the first few minutes, the vapor deposition released the chemical progressively over time (Figure 6A). To avoid a huge local concentration of NVP during in vivo experiments, we decided, based on our previous expertise, to use the 10% vapor deposition condition. Moreover, the surface of treated membranes examined by scanning electron microscopy (SEM) showed that the structure and integrity were preserved with the vapor deposition technique (Figure 6B) and was thus more suitable for in vivo analysis.

### 2.4. NVP Enhances Bone Regeneration In Vivo

**Figure 7:**
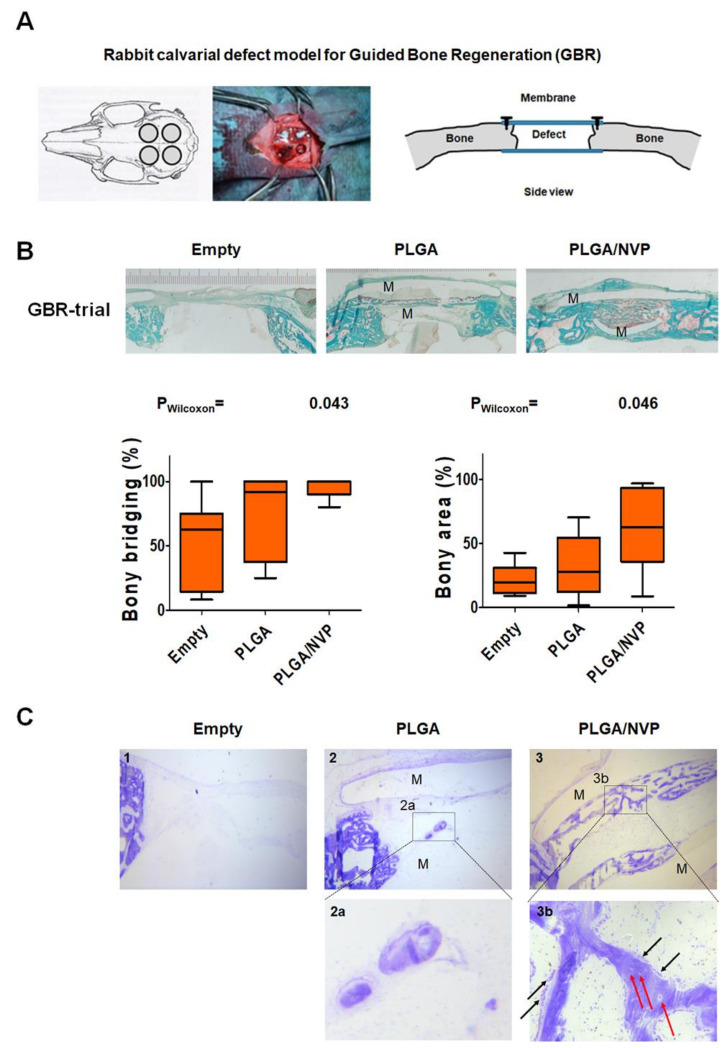
In vivo experiment with a NVP-loaded membrane. (**A**) Rabbit calvarial defect model for Guided Bone Regeneration (GBR). (**B**) Six New Zealand white rabbits were used in this study (*n* = 6). Goldner’s Trichrome staining and analysis of bone regeneration parameters of calvarial defects, control defect (Empty), defect treated with native PLGA membranes (PLGA), and defect treated with PLGA membranes loaded with NVP at 10% by vapor deposition (PLGA/NVP). **M** marks the transparent layer of the membranes placed above and below the defect. Trichrome stains the bone in a greenish-blue color and the osteoid in red. Results for bony bridging and bony regenerated area are displayed as box plots ranging from the 25th (lower quartile) to the 75th (upper quartile) percentile, including the median as a solid black line and whiskers showing the minimum and maximum values (*p* values are provided). (**C**) Histology of the calvaria defect. The micrographs show nondemineralized toluidine blue-stained sections of calvaria defects. There is no visible bone formation when the defect is left untreated (**1**). In the presence of a native PLGA membrane, there is no obvious bone formation in the middle of the defect except a bone-like structure with no visible osteoblast (**2, 2a**). However, bone formation begins on both sides of the defect. A significant bone formation is observed on the whole defect area when the NVP loaded membranes are used to cove the defect (**3**). At high magnification, the presence of bone-forming surface with a wide unmineralized osteoid seam lined by osteoblasts (black arrows in **3b**) as well as osteocytes (red arrows in **3b**). [Magnification. (**1**–**3**): 20×; (2**a**,3**b**): 40×].

To investigate the effect of NVP on bone regeneration, we used a guided bone regeneration model and delivered NVP via a biodegradable membrane. The size of the membranes applied was set at 2 × 38 mm^2^ and the volume surrounded by the two membranes was set at 100 µL. Based on these estimates, 10% vapor deposition membranes did not exceed 10 mM, the highest non-toxic dose used in our in vitro experiments. Therefore, only a 10% vapor deposition membrane was chosen for the in vivo experiment. The rabbit calvarial defect model used in our study for guided bone regeneration is illustrated (Figure 7A). Four weeks after the operations, the animals were sacrificed and the calvarias were collected and processed for histological analysis. Representative histology images of the middle sections are presented in Figure 7B. Histomorphometric analysis, based exclusively on the middle-section of the defect, showed a significantly higher bony bridging achieved by the application of the 10% vapor deposition membrane (PLGA/NVP) compared to the empty control defect. No significant difference was seen with the native PLGA membrane. However, the bone area achieved with PLGA/NVP membrane was significantly higher compared to the unloaded membrane (PLGA). The toluidine blue stained sections presented in Figure 7C show that, in all conditions, the onset of bone formation is observed at the ends of the defects. However, bone formation in the middle of the defects is only visible when membranes loaded with NVP cover the defect. At high magnification, the presence of bone-forming surface with a large non-mineralized osteoid layer surrounded by osteoblasts (black arrows in b) as well as osteocytes (red arrows in b).

## 3. Discussion

Over the past years, we have shown that NMP and DMA, two excipients approved by the FDA and used in the pharmaceutical industry, have intrinsic biological effects and can no longer be considered inactive small chemicals [10,30]. These two small active chemicals are able to increase the effect of BMP2 on osteoblast differentiation in vitro and bone regeneration in vivo. In addition, as inhibitors of osteoclast differentiation, they have a positive effect on the bone remodeling cycle. The effects of NMP and DMA may be related, to some extent, to their potential to regulate the epigenetic process by acting as bromodomain (BRD) inhibitors [7,8,31]. In terms of chemical structure, NMP and DMA resemble the acetyl group (CH_3_CO) and therefore mimic the acetylated lysine and prevent the binding of bromodomain containing protein to histone tail causing changes in gene transcription [32].

In this study, we investigated the effect of NVP, another organic solvent with a similar structure to NMP. In fact, the chemical structure of NVP suggested that this chemical could also interfere in the binding of protein-containing BRD domains with the acetylated lysine of histones or scaffolding proteins. Using an AlphaScreening assay, we demonstrated that NVP has the potential to be a ligand for bromodomains and thus act as a BRD inhibitor (Figure 1). By targeting protein expression at the epigenetic level, some therapeutic agents have shown promising anti-tumor activity and have already been tested in clinical trials [32,33]. Recent studies have shown that JQ1, a potent inhibitor of BRD2, BRD3 and BRD4 among other proteins, suppresses inflammation and bone destruction by inhibiting osteoclast maturation [34,35].

The fact of having used C2C12 multipotent cells, lacking the autologous expression of BMP2, allowed us to demonstrate that NVP acts as a synergistic factor (for BMP2) and not as a factor stimulating osteoblastic differentiation. Indeed, NVP alone cannot direct C2C12 cells to the osteoblastic lineage. However, in combination with BMP2, NVP markedly enhances the osteogenic effect of this growth factor (Figure 2).

At the cellular level, BMP2 binds to serine-threonine transmembrane receptors and induces a series of events that transduce the signal to downstream genes [36,37,38]. BMPs signal by phosphorylation of Smads molecules that translocate to the nucleus when activated and regulate the transcription of specific target genes. BMP2-Smad1/5/9 signaling is a known mediator of Runx2 expression [39]. Our results show that NVP increases the nuclear translocation of phosphorylated Smad1/5/9 induced by BMP2 (Figure 3) and thus acts as an enhancer of the osteogenic effect of BMP-2.

At high doses, BMP2 mediates harmful effects [40]. Thus, lowering BMP2 doses when combined with NVP provides a way to overcome the harmful effects of BMP2. In this regard, we would like to mention that NVP per se did not have any toxic effect at the concentrations used in this study.

The other aspect to be considered in the perspective of successful bone regeneration is the capacity of bone tissue to remodel itself and to closely align in quality and quantity to the original tissue. Bone remodeling is a dynamic and coordinated process that is maintained by a balance between bone formation and bone resorption [7].

In this study, in addition to its role in osteoblast differentiation, we have described that NVP is able to inhibit osteoclast differentiation in vitro as measured by TRAP activity (Figure 4). NVP decreases the number of nuclei per MNC suggesting that NVP inhibits the fusion of the mononuclear precursor cells. NFATc1 and c-Fos are the key transcription factors in RANKL-induced osteoclast differentiation. The role of the c-Fos has been shown by knockout experiments [25]. C-Fos knockout mice display a severe osteopetrotic phenotype due to a failure to form osteoclasts. In addition, previous reports established that NFATc1 is not induced by RANKL in osteoclasts lacking c-Fos, and NFATc1 knockout mouse-derived stem cells failed to differentiate into osteoclasts [25,41]. In our study, NVP significantly suppressed RANKL-induced NFATc1 and c-Fos mRNA and protein expression (Figure 5). It is well known that the AP-1 complex, which is a dimeric transcription factor composed of members of the Jun and Fos protein family, regulates NFATc1. AP-1 transforms extracellular signals in bone and immune cells into changes in the expression of specific target genes that harbor an AP-1-binding site(s) in their promoter or enhancer region [41]. Our results suggests that the suppression of NFATc1 expression by NVP is the consequence of the down-regulation of c-Fos, with potentially the subsequent down-regulation of AP-1 activity. Cathepsin K is responsible for the breakdown of type I collagen and is generally considered to be the main bone-degrading enzyme. Moreover, Cathepsin K is required for the initial formation of actin rings and thus for the activation of osteoclasts [42]. In this study, we showed that NVP inhibited the RANKL-induced up-regulation of cathepsin K mRNA suggesting that NVP play a critical role in the actin ring formation process and therefore in inhibiting bone resorption.

By controlling both the osteoblasts and osteoclasts differentiation, NVP demonstrates to be a useful candidate for bone regeneration procedure. In order to repair a bone defect or augment alveolar bone for dental implant treatment, guided bone regeneration (GBR) procedures have frequently been performed. In addition to providing a barrier function, the membranes used in GBR procedures should be biocompatible, offer appropriate mechanical properties and ideally must be bioactive to promote tissue regeneration. In previous studies, we have used two small molecules to make membranes bioactive. Indeed, by treating membranes with NMP or DMA, which were capable of increasing the osteoblastic differentiation induced by BMP2 and therefore bone formation; we have been able to demonstrate the superiority of these membranes compared to untreated ones [19,28]. By analyzing cell differentiation in vitro, it was found that NVP was more active than NMP or DMA in increasing the differentiation of osteoblasts induced by BMP2. We therefore wanted to know if membranes treated with NVP could also increase bone regeneration in vivo, as already shown with NMP and DMA [28,31]. In vivo data indicated that NVP added bioactivity to the membranes, as showed by the increased bony bridging and bony regenerated area (Figure 7). The results presented in this study indicate that in addition to its effect on several markers of osteoblastic and osteoclastic cell differentiation, NVP promotes bone regeneration in vivo and may be a potential therapeutic option in guided bone regeneration procedures. NVP adds bioactivity to PLGA membranes and a major advantage of its use would likely be the treatment of more challenging defects in combination with BMP2. This could both increase the activity of BMP2 while helping to decrease its concentrations, which is harmful at high doses.

Another area where NVP may be useful is the treatment or prevention of osteoporosis, most common metabolic disease. Osteoporosis is a skeletal disorder characterized by a reduction of bone strength and increased risk of bone fracture.

**Figure 8:**
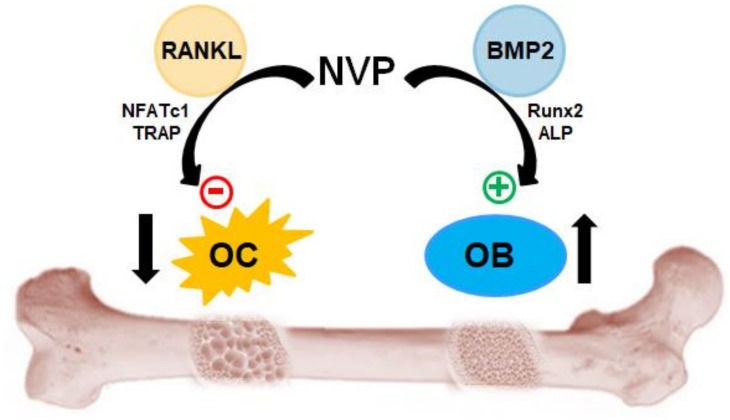
Role of NVP in bone homeostasis. NVP has a dual function in the inhibition of RANKL-induced osteoclastogenesis and the stimulation of BMP2-induced osteogenesis.

Several factors impact bone homeostasis, including heredity, gender, endocrine status as well as sporadic risk factors such as smoking. In addition to these modifiable factors, the contribution of epigenetic regulation of genes in many diseases has been documented, making it a crucial target for basic and clinical research for multifactorial diseases, including osteoporosis [43,44]. In this sense, we have shown in in vivo studies that NMP and DMA, both bromodomain inhibitors, could effectively protect against loss of bone density induced by estrogen depletion [8,30]. Since NVP has high homology with NMP and is also used as an excipient in the pharmaceutical industry, we could reasonably assume that NVP could be as effective as NMP or DMA. However, additional in vitro and in vivo experiments are required to identify more precisely the mode of action of NVP. Nonetheless, the results presented in this study suggest that NVP plays a significant role in bone homeostasis and could be a promising therapeutic substance for bone metabolic disorders including osteoporosis (Figure 8).

In conclusion, our in vitro studies show that NVP functions as bromodomain inhibitor, BMP2 enhancer and osteoclast differentiation inhibitor. Moreover, the small chemical NVP significantly enhanced bone regeneration in vivo. In light of all these effects, NVP is a promising candidate as a direct or adjuvant therapeutic compound for bone tissue regeneration and engineering.

## 4. Materials and Methods

### 4.1. Reagents and Antibodies

Dulbecco’s modified Eagle’s medium GlutaMAX (DMEM), fetal bovine serum (FBS), penicillin, streptomycin, protease and phosphatase inhibitors cocktail were purchased from Thermo Fisher Scientific (Waltham, MA, USA). Recombinant human sRANKL (Invitrogen_PHP0034) was purchased from Thermo Fisher Scientific. Anti-NFATc1 (sc-7294) and anti-c-Fos (sc-166940) polyclonal antibodies were obtained from Santa Cruz Biotechnology Inc. (Santa Cruz, CA, USA). Anti-phospho-Smad1/5/9 and anti-GAPDH antibodies were obtained from Cell Signaling (Cell Signaling Technology, Danvers, MA, USA). Primers for RT-PCR (QuantiTect primer assay, Table S1) and RNA extraction kit (RNeasy kit) were purchased from Qiagen (Hilden, Germany). The primers for Cathepsin K and c-Fos were designed as already described [10] and ordered from Microsynth (Balgach, Switzerland). TRAP staining solution (leukocyte acid phosphatase kit, 386-A) was purchased from Sigma. All others chemicals were obtained from Sigma (St. Louis, MO, USA).

### 4.2. AlphaScreening Assay

The AlphaScreening assay was performed as previously described [8]. Briefly, this assay was performed using recombinant bromodomains and bromodomain ligands OR recombinant BET bromodomains and BET Ligand from BPS Bioscience (San Diego, CA, USA). The AlphaScreening signal from the assay is correlated with the amount of bromodomain/BET ligand binding to the bromodomain. In the absence of the compound, the AlphaScreening signal (At) in each data set was defined as 100% activity. In the absence of the bromodomain/BET Ligand, the AlphaScreening signal (Ab) in each data set was defined as 0% activity. The percent activity in the presence of each compound was calculated according to the following equation:

% activity = [(A − Ab)/(At − Ab)] × 100

where A = AlphaScreening signal in the presence of the compound, Ab = AlphaScreening signal in the absence of the bromodomain/BET Ligand, and At = AlphaScreening signal in the absence of the compound.

### 4.3. Cell Cultures

C2C12 and RAW264.7 cell lines were purchased from the American Type Culture Collection (ATCC, Manassas, VA, USA). Cells were cultured in DMEM supplemented with 10% FBS and antibiotics (100 U/mL penicillin G and 100 mg/mL streptomycin). The cultures were never allowed to become confluent. The cells were grown at 37 °C in humidified air mixed with 5% CO_2_. For the experiments, the cells were plated one day before treatment and treated with BMP2 (C2C12 cells) or RANKL (RAW264.7 cells) in the presence or absence of different concentration of NVP.

### 4.4. Primary Rabbit Osteoblast

The local authorities (Veterinäramt Zurich, Switzerland) have approved all animal experiments (licenses 115/2015 and 065/2018). Calvaria from two-month-old New Zealand rabbits were used for the isolation of osteoblasts by enzymatic digestion. In detail, after several washing steps in PBS, the calvaria were cut into pieces of the size of 1.0 to 2.0 mm^3^. Then the tissue was digested with 0.1% (*v/v*) type 2 collagenase (Worthington, Lakewood, NJ, USA) at 37 °C, 5% CO_2_ for 30 min. After digestion, the suspension was passed through a 100 µm cell strainer to remove undigested tissue. After centrifugation at 1500 rpm for 5 min, cells were resuspended in alpha-minimum essential medium (α-MEM) containing 10% (*v/v*) fetal bovine serum (FBS), 1% (*v/v*) penicillin/streptomycin/glutamine and 1% HEPES.

For osteogenic differentiation, osteoblasts were seeded in a 24-well plate and grown to 90% confluence. The cells were then incubated in a differentiation medium (osteogenic medium composed of α-MEM supplemented with 1% P/S, 10% FCS, 100 nM dexamethasone, 10 mM β-glycerophosphate and 50 μM l-ascorbic acid-2-phosphate (all from Sigma-Aldrich, Saint-Louis, MO, USA). The culture was maintained at 37 °C, 5% CO_2_ environment, and the medium was renewed twice a week. The differentiation test was carried out with two technical duplicates.

### 4.5. Primary Bone Marrow-Derived Monocytes (BMMs)

Bone marrow-derived macrophages (BMMs) were isolated from the long bones of—two-month-old New Zealand rabbits and were maintained in α-minimal essential medium containing 10% heat-inactivated FBS in the presence of M-CSF (100 ng/mL) as described previously [10]. To generate osteoclasts from BMMs, cells were plated in 12-well tissue culture plates and cultured in the presence of 25 ng/mL RANKL and 25 ng/mL M-CSF.

### 4.6. Cell Viability and Proliferation Assay

The effect of different concentrations of NVP on cell viability/toxicity was analyzed using a nonradioactive WST-1 cell proliferation assay kit (Roche Diagnostics, Basel, Switzerland) according to the manufacturer’s instructions.

### 4.7. Assay of ALP Activity and ALP Staining

Alkaline phosphatase activity (ALP) was measured as a marker of osteoblastic differentiation. After cell stimulation, ALP activity was measured using *p*-nitrophenylphosphate (Sigma, Saint-Louis, MO, USA) as a substrate as previously described [45]. To examine ALP histochemically, cells were fixed for 10 min with 3.7% formaldehyde at room temperature. After washing with PBS, the cells were stained as previously described [22]. Image of stained cells were captured with a CDD camera.

### 4.8. Assay of TRAP Activity and TRAP Staining

RAW264.7 cells were plated in a 24-well culture dish with different concentrations of NVP in the presence of 25 ng/mL RANKL. After 6 days of culture, TRAP activity in the cell lysate was measured as previously described [10].

TRAP histochemical staining was performed using a leukocyte acid phosphatase kit (Sigma). Cultured cells were fixed with formaldehyde for 10 min at room temperature, washed with PBS, and air-dried. After TRAP staining, TRAP-positive multinucleated cells (i.e., more than three nuclei) were counted under a phase-contrast microscope.

### 4.9. Quantitative Real-Time Polymerase Chain Reaction

RNA was extracted using the RNeasy kit (Qiagen). RNA quantity and quality were determined using Nanodrop 2000 Spectrophotometer. Reverse transcription of 1µg RNA was performed using iScript™ Reverse Transcription Supermix according to manufacturer’s recommendations (BioRad, Hercules, CA, USA). The polymerase chain reaction was realized with each sample in duplicates using a Bio-Rad CFX96 Real-Time System and SYBR^®^ Green Supermix (BioRad, Hercules, CA, USA) using specific primers. Gene expression was normalized to the reference gene ribosomal protein s18 (rps18) or glyceraldehyde 3-phosphate dehydrogenase (GAPDH) using the comparative ∆∆C_T_ method. The primers used in this study were commercially available and obtained from Qiagen (Table S1) or ordered from Microsynth (Microsynth, Switzerland) as already described [10].

### 4.10. Protein Preparation and Western Blot Analysis

The treated cells were rapidly frozen in liquid nitrogen and stored at −80 °C for further analysis. Cells were lysed as described previously [10]. Proteins were fractionated onto a 4–20% SDS-polyacrylamide gel, transferred to a PVDV membrane using precast Trans-Blot Turbo stack (Bio Rad, Hercules, CA, USA), and immunoblotted with specific antibodies (Table S2). Detection was performed using Clarity Western Enhanced Chemiluminescence (ECL) substrate in conjunction with the ChemiDoc MP imaging system (Bio Rad).

### 4.11. Immunofluorescence Staining, pSmad1/5/9 Nuclear Translocation

C2C12 cells were grown on 15 µ-Chamber 12 well (Ibidi, Martinsried, Germany), stimulated with 100 ng/mL of BMP2 alone (BMP2) or BMP2 after 1h of NVP pre-treatment (BMP+NVP). After 60 min of BMP2 stimulation, cells were fixed in 4 % paraformaldehyde for 15 min at room temperature. Next, the subcellular localization of the pSmad1/5/9 was shown by immunofluorescence staining. The cytoskeleton (actin filaments) was labelled with DyLight554 Phalloidin (CST, Danvers, MA, USA). The ProLong Gold Antifade Reagent with DAPI (CST) was used as a mounting reagent and counterstaining.

### 4.12. NVP Loading of PLGA Membranes

PLGA membranes (Inion GTR^TM^ membranes, Inion Oy, Tampere, Finland) were loaded with pure NVP by either a vapor deposition method (V-NVP) or direct dipping (D-NVP) into the liquid chemical as previously described [19]. Briefly, vapor deposition was performed at room temperature in a desiccator connected to a vacuum pump. Inside the desiccator, the membranes were placed onto a metal mesh laying over a glass dish filled with pure NVP. Dip loading of the membrane was achieved by dipping the membrane into pure NVP for 10 s.

### 4.13. In Vitro Release of NVP

The release experiment was performed in triplicate. Equal-sized, chemical-loaded membrane samples were placed into glass bottles containing 50 mL of phosphate-buffered saline (PBS, pH 7.4). For the time course of the in vitro release, samples were kept agitating at 37 °C. At indicated time points, 200 µL were removed from the sample and stored at 4 °C for subsequent analysis. NVP concentration was measured at 220 nm, using 96-well plates coated to enable reading at UV wavelengths (Corning, Corning, NY, USA). Values were compared to a standard curve of known NVP concentrations. To estimate the non-toxic concentration of the released NVP for the in vivo experiments, the membrane surface and release volume were taken into consideration.

### 4.14. Scanning Electron Microscopy for Structural Analysis

NVP-loaded membranes were air-dried, fixed onto metal stubs, and gold-coated using a gold sputter machine (SCD 030, Baltec, Balzers, Liechtenstein) as previously described [19]. The membrane structure was analyzed using the Zeiss Supra V50 SEM (Carl Zeiss, Oberkochen, Germany) at an acceleration voltage of 5 kV. Access to the SEM was kindly provided by the Center for Microscopy and Image Analysis (ZMB) at the University of Zürich.

### 4.15. Animal Model for Bone Regeneration

The local authorities (Veterinäramt Zurich, Switzerland) approved all animal experiments (licenses 115/2015 and 065/2018), which are in line with the EU Directive 2010/63/EU for animal experiments. Six New Zealand white rabbits were used in this study. Animal weights ranged from 3.5 to 4.0 kg, and all animals were fed a standard laboratory diet. Prior to surgery, animals were anesthetized by injection of 65 mg/kg ketamine and 4 mg/kg xylazine. Anesthesia was maintained with isoflurane/O_2_. The creation of defects and the guided bone regeneration procedures were described previously [19]. Four weeks after the surgery, rabbits received general anesthesia and were sacrificed by an overdose of pentobarbital. The cranial section containing the craniotomy sites was removed and processed for histology as previously reported [19]. It is important to mention that, in an effort to reduce the number of animals, we tested two molecules in parallel and therefore the controls were used to analyze both studies. In the initial experiment that was published last year, we reported that one of the defects was left without a membrane (empty control defect), one with a native PLGA membranes (membranes no chemicals), one with a membrane loaded with DMA (PLGA/DMA) and finally the last defect with a membranes loaded with an unknown substance (in this case NVP). The results for empty defects and those for the untreated membranes were used as controls for both studies.

### 4.16. Histology

Excised calvarial bone samples were fixed with 70% ethanol and dehydrated using a sequential water substitution process. Fixation and defatting were allowed for 72 h in xylene and plastic infiltration was performed using methyl methacrylate (MMA, Sigma M55909). Samples were then embedded in MMA containing 0.5% Perkadox 16 (Dr. Grogg Chemie AG, Stettlen, Switzerland, G425), 15% dibuthylthalate (Sigma-Aldrich, St. Louis, MO, USA, 524980), and 0.01% Pentaerythritol tetrakis (Sigma-Aldrich 441783). Fully polymerized samples were cut using a Mecatome T180 (Presi, Le Locle, Switzerland) and further processed to 10 µm sections (Leica Reichert Jung Polycut S, Austin, TX, USA). Sections from the middle of the samples were stained using Goldner’s trichrome method. Images were acquired using a Leica microscope with a millimeter-scale and were analyzed using the Adobe Photoshop program.

For toluidine blue staining, the sections were de-plasticized by three changes of 2-methylethylacetate for 20 min each, two changes of acetone for 5 min each, and two changes of deionized water for 5 min each. Sections were stained with toluidine blue as previously described [19].

### 4.17. Statistical Analysis

Experiments were carried out independently at least three times. Results are expressed as the mean ± S.D. To assess the effects of treatment and time on cells, one-way ANOVA tests was performed with a Bonferroni’s post-hoc tests. Results were considered significantly different for *p* < 0.05. For the in vivo results, the significance was determined with the non-parametric Wilcoxon-signed rank test.

## Data Availability

The data presented in this study are available on request from the corresponding authors.

## Supplementary Materials

The following are available online at https://www.mdpi.com/article/10.3390/ijms222011052/s1.

## References

[1] Haaf F., Sanner A., Straub F. (1985). Polymers of N-Vinylpyrrolidone: Synthesis, Characterization and Uses. Polym. J..

[2] Moffitt E.A. (1975). Blood substitutes. Can. Anaesth. Soc. J..

[3] Luo Y., Hong Y., Shen L., Wu F., Lin X. (2021). Multifunctional Role of Polyvinylpyrrolidone in Pharmaceutical Formulations. AAPS PharmSciTech.

[4] Poulsen E. (1991). Safety evaluation of substances consumed as technical ingredients (food additives). Food Addit. Contam..

[5] Waleka E., Stojek Z., Karbarz M. (2021). Activity of Povidone in Recent Biomedical Applications with Emphasis on Micro- and Nano Drug Delivery Systems. Pharmaceutics.

[6] Florencio-Silva R., Sasso G.R., Sasso-Cerri E., Simoes M.J., Cerri P.S. (2015). Biology of Bone Tissue: Structure, Function, and Factors That Influence Bone Cells. Biomed. Res. Int..

[7] Ghayor C., Weber F.E. (2016). Epigenetic Regulation of Bone Remodeling and Its Impacts in Osteoporosis. Int. J. Mol. Sci.

[8] Gjoksi B., Ghayor C., Siegenthaler B., Ruangsawasdi N., Zenobi-Wong M., Weber F.E. (2015). The epigenetically active small chemical N-methyl pyrrolidone (NMP) prevents estrogen depletion induced osteoporosis. Bone.

[9] Chen T.H., Weber F.E., Malina-Altzinger J., Ghayor C. (2019). Epigenetic drugs as new therapy for tumor necrosis factor-alpha-compromised bone healing. Bone.

[10] Ghayor C., Correro R.M., Lange K., Karfeld-Sulzer L.S., Gratz K.W., Weber F.E. (2011). Inhibition of osteoclast differentiation and bone resorption by N-methylpyrrolidone. J. Biol. Chem..

[11] Boyson S.P., Gao C., Quinn K., Boyd J., Paculova H., Frietze S., Glass K.C. (2021). Functional Roles of Bromodomain Proteins in Cancer. Cancers.

[12] Gong F., Chiu L.Y., Miller K.M. (2016). Acetylation Reader Proteins: Linking Acetylation Signaling to Genome Maintenance and Cancer. PLoS Genet..

[13] Mirguet O., Lamotte Y., Chung C.W., Bamborough P., Delannee D., Bouillot A., Gellibert F., Krysa G., Lewis A., Witherington J. (2014). Naphthyridines as novel BET family bromodomain inhibitors. Chem. Med. Chem..

[14] Zeng L., Li J., Muller M., Yan S., Mujtaba S., Pan C., Wang Z., Zhou M.M. (2005). Selective small molecules blocking HIV-1 Tat and coactivator PCAF association. J. Am. Chem. Soc..

[15] Filippakopoulos P., Qi J., Picaud S., Shen Y., Smith W.B., Fedorov O., Morse E.M., Keates T., Hickman T.T., Felletar I. (2010). Selective inhibition of BET bromodomains. Nature.

[16] Benic G.I., Hammerle C.H. (2014). Horizontal bone augmentation by means of guided bone regeneration. Periodontol. 2000.

[17] Esposito M., Grusovin M.G., Felice P., Karatzopoulos G., Worthington H.V., Coulthard P. (2009). Interventions for replacing missing teeth: Horizontal and vertical bone augmentation techniques for dental implant treatment. Cochrane Database Syst. Rev..

[18] Miguel B.S., Ghayor C., Ehrbar M., Jung R.E., Zwahlen R.A., Hortschansky P., Schmoekel H.G., Weber F.E. (2009). N-methyl pyrrolidone as a potent bone morphogenetic protein enhancer for bone tissue regeneration. Tissue Eng. Part A.

[19] Siegenthaler B., Ghayor C., Ruangsawasdi N., Weber F.E. (2020). The Release of the Bromodomain Ligand N,N-Dimethylacetamide Adds Bioactivity to a Resorbable Guided Bone Regeneration Membrane in a Rabbit Calvarial Defect Model. Materials.

[20] Gjoksi B., Ghayor C., Bhattacharya I., Zenobi-Wong M., Weber F.E. (2016). The bromodomain inhibitor N-methyl pyrrolidone reduced fat accumulation in an ovariectomized rat model. Clin. Epigenet..

[21] Gjoksi B., Ruangsawasdi N., Ghayor C., Siegenthaler B., Zenobi-Wong M., Weber F.E. (2017). Influence of N-methyl pyrrolidone on the activity of the pulp-dentine complex and bone integrity during osteoporosis. Int. Endod. J..

[22] Katagiri T., Yamaguchi A., Komaki M., Abe E., Takahashi N., Ikeda T., Rosen V., Wozney J.M., Fujisawa-Sehara A., Suda T. (1994). Bone morphogenetic protein-2 converts the differentiation pathway of C2C12 myoblasts into the osteoblast lineage. J. Cell Biol..

[23] Pennypacker B., Shea M., Liu Q., Masarachia P., Saftig P., Rodan S., Rodan G., Kimmel D. (2009). Bone density, strength, and formation in adult cathepsin K (-/-) mice. Bone.

[24] Ishida N., Hayashi K., Hoshijima M., Ogawa T., Koga S., Miyatake Y., Kumegawa M., Kimura T., Takeya T. (2002). Large scale gene expression analysis of osteoclastogenesis in vitro and elucidation of NFAT2 as a key regulator. J. Biol. Chem..

[25] Matsuo K., Galson D.L., Zhao C., Peng L., Laplace C., Wang K.Z., Bachler M.A., Amano H., Aburatani H., Ishikawa H. (2004). Nuclear factor of activated T-cells (NFAT) rescues osteoclastogenesis in precursors lacking c-Fos. J. Biol. Chem..

[26] Takayanagi H., Kim S., Koga T., Nishina H., Isshiki M., Yoshida H., Saiura A., Isobe M., Yokochi T., Inoue J. (2002). Induction and activation of the transcription factor NFATc1 (NFAT2) integrate RANKL signaling in terminal differentiation of osteoclasts. Dev. Cell.

[27] Khorsand B., Elangovan S., Hong L., Kormann M.S.D., Salem A.K. (2019). A bioactive collagen membrane that enhances bone regeneration. J. Biomed. Mater. Res. B Appl. Biomater..

[28] Karfeld-Sulzer L.S., Ghayor C., Siegenthaler B., Gjoksi B., Pohjonen T.H., Weber F.E. (2017). Comparative study of NMP-preloaded and dip-loaded membranes for guided bone regeneration of rabbit cranial defects. J. Tissue Eng. Regen. Med..

[29] Karfeld-Sulzer L.S., Ghayor C., Siegenthaler B., de Wild M., Leroux J.C., Weber F.E. (2015). N-methyl pyrrolidone/bone morphogenetic protein-2 double delivery with in situ forming implants. J. Control. Release.

[30] Ghayor C., Gjoksi B., Dong J., Siegenthaler B., Caflisch A., Weber F.E. (2017). N,N Dimethylacetamide a drug excipient that acts as bromodomain ligand for osteoporosis treatment. Sci. Rep..

[31] Siegenthaler B., Ghayor C., Gjoksi-Cosandey B., Ruangsawasdi N., Weber F.E. (2018). The Bromodomain Inhibitor N-Methyl pyrrolidone Prevents Osteoporosis and BMP-Triggered Sclerostin Expression in Osteocytes. Int. J. Mol. Sci..

[32] Shortt J., Hsu A.K., Martin B.P., Doggett K., Matthews G.M., Doyle M.A., Ellul J., Jockel T.E., Andrews D.M., Hogg S.J. (2014). The drug vehicle and solvent N-methylpyrrolidone is an immunomodulator and antimyeloma compound. Cell Rep..

[33] Prinjha R.K., Witherington J., Lee K. (2012). Place your BETs: The therapeutic potential of bromodomains. Trends Pharmacol. Sci..

[34] Lamoureux F., Baud’huin M., Rodriguez Calleja L., Jacques C., Berreur M., Redini F., Lecanda F., Bradner J.E., Heymann D., Ory B. (2014). Selective inhibition of BET bromodomain epigenetic signalling interferes with the bone-associated tumour vicious cycle. Nat. Commun..

[35] Meng S., Zhang L., Tang Y., Tu Q., Zheng L., Yu L., Murray D., Cheng J., Kim S.H., Zhou X. (2014). BET Inhibitor JQ1 Blocks Inflammation and Bone Destruction. J. Dent. Res..

[36] Celil A.B., Hollinger J.O., Campbell P.G. (2005). Osx transcriptional regulation is mediated by additional pathways to BMP2/Smad signaling. J. Cell Biochem..

[37] Drissi M.H., Li X., Sheu T.J., Zuscik M.J., Schwarz E.M., Puzas J.E., Rosier R.N., O’Keefe R.J. (2003). Runx2/Cbfa1 stimulation by retinoic acid is potentiated by BMP2 signaling through interaction with Smad1 on the collagen X promoter in chondrocytes. J. Cell Biochem..

[38] Wang R.N., Green J., Wang Z., Deng Y., Qiao M., Peabody M., Zhang Q., Ye J., Yan Z., Denduluri S. (2014). Bone Morphogenetic Protein (BMP) signaling in development and human diseases. Genes Dis..

[39] Lee K.S., Hong S.H., Bae S.C. (2002). Both the Smad and p38 MAPK pathways play a crucial role in Runx2 expression following induction by transforming growth factor-beta and bone morphogenetic protein. Oncogene.

[40] James A.W., LaChaud G., Shen J., Asatrian G., Nguyen V., Zhang X., Ting K., Soo C. (2016). A Review of the Clinical Side Effects of Bone Morphogenetic Protein-2. Tissue Eng. Part B Rev..

[41] Wagner E.F., Eferl R. (2005). Fos/AP-1 proteins in bone and the immune system. Immunol. Rev..

[42] Wilson S.R., Peters C., Saftig P., Bromme D. (2009). Cathepsin K activity-dependent regulation of osteoclast actin ring formation and bone resorption. J. Biol. Chem..

[43] Chen W., Zhang B., Chang X. (2021). Emerging roles of circular RNAs in osteoporosis. J. Cell Mol. Med..

[44] Xu Y., Ma J., Xu G., Ma D. (2021). Recent advances in the epigenetics of bone metabolism. J. Bone Miner. Metab..

[45] Ghayor C., Ehrbar M., San Miguel B., Gratz K.W., Weber F.E. (2009). cAMP enhances BMP2-signaling through PKA and MKP1-dependent mechanisms. Biochem. Biophys. Res. Commun..

