# Supplementary material for: Effect of N-Vinyl-2-Pyrrolidone (NVP), a Bromodomain-Binding Small Chemical, on Osteoblast and Osteoclast Differentiation and Its Potential Application for Bone Regeneration
Source: Int J Mol Sci. 2021 Oct 13;22(20):11052. doi: 10.3390/ijms222011052 (PMC8541071; doi:10.3390/ijms222011052)
Supplement: Supplementary file 1 [file ijms-22-11052-s001.zip › ijms-1360146-supplementary.pdf]

•Molecular Formula $C_5H_9NO$

•Average mass 99.131 Da

**N-methylpyrrolidone (NMP)**

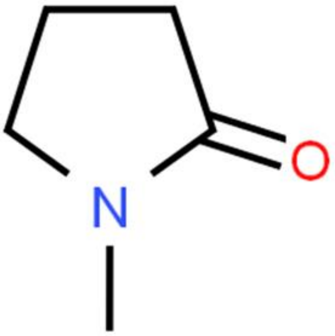


•Molecular Formula $C_6H_9NO$

•Average mass 111.142 Da

**1-Vinyl-2-pyrrolidone (NVP)**

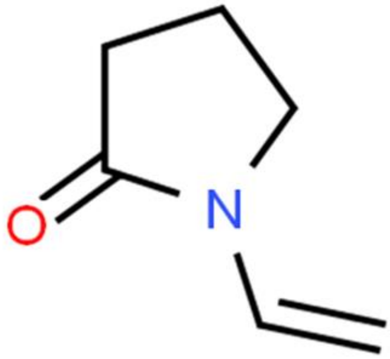


**Figure S1**: chemical structures of NMP and NVP

| Gene | Species | Company | Catalog Nr. |
|---|---|---|---|
| ALP | Mouse | QIAGEN | QT00157717 |
| Runx2 | Mouse | QIAGEN | QT00102193 |
| TRAP | Mouse | QIAGEN | QT00131012 |
| NFATc1 | Mouse | QIAGEN | QT00167692 |
| GAPDH | Mouse | QIAGEN | QT01658692 |
| Rsp18 | Mouse | QIAGEN | QT02448075 |

**Table S1**: Primers used in qRT-PCR experiments.

| Antibody | Company | Catalog Nr. |
|---|---|---|
| GAPDH | Cell Signaling Technology | #5174 |
| pSmad 1/5/9 | Cell Signaling Technology | #13820 |
| Anti-rabbit IgG HRP-linked | Cell Signaling Technology | #7074 |
| NFATc1 | Santa Cruz Biotechnology | Sc-7294 |
| c-Fos | Santa Cruz Biotechnology | Sc-398595 |

**Table S2**: Antibodies used in Western blot and immunofluorescence experiments
